# ACPred-BMF: bidirectional LSTM with multiple feature representations for explainable anticancer peptide prediction

**DOI:** 10.1038/s41598-022-24404-1

**Published:** 2022-12-19

**Authors:** Bingqing Han, Nan Zhao, Chengshi Zeng, Zengchao Mu, Xinqi Gong

**Affiliations:** 1 Institute for Mathematical Sciences, Renmin University of China, Beijing, 100872 China; 2 School of Mathematics and Statistics, Shandong University, Weihai, 264209 China; 3 Beijing Academy of Artificial Intelligence, Beijing, 100083 China

**Keywords:** Computational biology and bioinformatics, Computational models, Machine learning

## Abstract

Cancer has become a major factor threatening human life and health. Under the circumstance that traditional treatment methods such as chemotherapy and radiotherapy are not highly specific and often cause severe side effects and toxicity, new treatment methods are urgently needed. Anticancer peptide drugs have low toxicity, stronger efficacy and specificity, and have emerged as a new type of cancer treatment drugs. However, experimental identification of anticancer peptides is time-consuming and expensive, and difficult to perform in a high-throughput manner. Computational identification of anticancer peptides can make up for the shortcomings of experimental identification. In this study, a deep learning-based predictor named ACPred-BMF is proposed for the prediction of anticancer peptides. This method uses the quantitative and qualitative properties of amino acids, binary profile feature to numerical representation for the peptide sequences. The Bidirectional LSTM network architecture is used in the model, and the attention mechanism is also considered. To alleviate the black-box problem of deep learning model prediction, we visualized the automatically extracted features and used the Shapley additive explanations algorithm to determine the importance of features to further understand the anticancer peptide mechanism. The results show that our method is one of the state-of-the-art anticancer peptide predictors. A web server as the implementation of ACPred-BMF that can be accessed via: http://mialab.ruc.edu.cn/ACPredBMFServer/.

## Introduction

Cancer is a major public health problem worldwide^1^. According to data released by the International Agency for Research on Cancer (IARC), an agency under the WHO, there were 9.96 million cancer deaths worldwide in 2020. Deaths from cancer will continue to grow, reaching 16.3 million in 2040, according to IARC forecasts. Conventional chemotherapy, radiotherapy, and surgical treatments of cancer mainly focus on mass cell killing without high specificity and often cause severe side effects and toxicities^2–4^. Traditional treatments have limited efficacy and damage normal cells. Ideally, anticancer therapy should destroy a range of cancer types, but not all healthy cells^5^.

Under such circumstances, it is urgent to develop new therapeutic approaches to treat cancer. Anticancer peptides (ACPs) show great potential in the treatment of cancer: they destroy cancer cells via apoptosis and necrosis and they can inhibit tumor growth through immunomodulation^2,5^. This anticancer mechanism is nonspecific for cancer types, and is general to different cancers^6^. In addition, healthy cells are electrically neutral, while cancer cells contain negatively charged components on their surfaces, and their membranes are more fluid ^5,7,8^. When the cationic ACP interacts with cancer cells, it causes destabilization and lysis of the cancer cell membrane without damaging normal cells^2,5,9^. Compared with chemotherapy or surgery, ACPs are thought to have at least the same efficacy, but with additional advantages in terms of safety. Because chemotherapy often has serious adverse effects, surgery presents additional risks to patients^10^. Compared with other molecules, short peptides are less immunogenic, and more stable in vitro; at the same time, since the main products of peptide metabolism are amino acids, such drugs generally have lower toxicity features^7,10^.

ACP drugs have low toxicity, stronger efficacy and specificity for cancer cells, and have become a new type of cancer treatment drugs^7,11^. Rapid and accurate identification of potential ACPs in a large number of proteins is of great significance for the development of new drugs, however, identification by experimental methods is time-consuming, expensive, and difficult to apply in a high-throughput manner^12^. In contrast, using computational methods can avoid the shortcomings of traditional methods and achieve high-throughput prediction of ACPs. Therefore, it is of great practical significance to study high-performance predictors of ACPs.

In recent years, much research has been done for the prediction of ACPs, and has made excellent progress. ACP prediction methods are mainly divided into traditional machine learning-based methods and deep learning-based methods. Traditional machine learning-based methods mainly rely on manually extracting features, which are obtained by converting each peptide sequence into a fixed-length numeric vector, and use classifiers such as support vector machine (SVM) and random forest (RF) for identifying ACPs. In 2013, Tyagi et al.^13^ first proposed a machine learning-based predictor AntiCP, which used features such as amino acid composition (AAC), dipeptide composition (DPC) and binary profile feature (BPF) as the input of the SVM classifier. In 2015, Vijayakumar et al.^14^ proposed the SVM-based predictor ACPP, and used protein relatedness measure which incorporates not only compositional information but also centroidal and distributional measures of amino acids. Subsequently, Chen et al.^15^ proposed the predictor called iACP developed by the approach of optimizing the g-gap dipeptide composition (g-gap DPC), which used the SVM classifier for prediction. In 2017, Akbar et al.^16^ developed an evolutionary intelligent genetic algorithm-based ensemble model called iACP-GAEnsC, in which the peptide sequences are formulated by three different features, i.e., amphiphilic pseudo amino acid composition (Am-PseAAC)^17^, g-gap DPC, and Reduced amino acid alphabet composition. In the same year, Balachandran et al.^18^ proposed MLACP, which describes peptide sequences based on features including AAC, DPC, atomic composition, and physicochemical properties, then uses SVM and RF for prediction. It can be seen that there are many types of sequence-based feature descriptors available, and feature selection is necessary to avoid the dimensional disaster and information redundancy. In 2018, Wei et al.^19^ developed an ACP prediction algorithm called ACPred-FL that extracts and learns a 40-dimensional feature vector from SVM-based models trained using sequence-based feature descriptors, and further through feature selection techniques to improve the feature representation ability. Rao et al.^20^ presented ACPred-Fuse that integrated a total of 29 different handcrafted features (HF) and performed feature selection on them. In 2019, Schaduangrat et al.^21^ proposed a predictor called ACPred, which is based on the feature combination of AAC, DPC, physicochemical properties, pseudo-amino acid composition (PseAAC)^22^, Am-PseAAC, etc., using RF and SVM as classifiers. Agrawal et al.^23^ proposed the predictor called AntiCP2.0, which was developed by extremely randomized trees (ETree) algorithm with the AAC and DPC. The traditional machine learning-based methods for ACP prediction have been very mature and have produced good prediction results on some datasets. However, the methods based on traditional machine learning themselves have some inherent defects. First, the feature extraction methods transform the peptide sequence into a fixed-length feature vector, which is easy to lose some information, especially for long sequences^24,25^. In addition, these feature extraction methods can only extract the local order of peptide sequence through features such as DPC^23^, and it is difficult to grasp the global order information. Finally, the performance of these methods is largely related to manual feature extraction mechanisms, but it is not easy to extract suitable features for different data^26^.

The deep learning-based methods for ACP prediction gradually came into existence in 2017^27^. Yi et al.^28^ proposed ACP-DL in 2019, which uses BPF and k-mer sparse matrix feature to represent the peptide sequences, and uses Long Short-Term Memory Model (LSTM) for prediction. Unlike traditional machine learning-based methods, deep learning-based methods do not require manual feature extraction to represent the input data^26^, that is, they can automatically extract features^29^. The methods based on deep learning can be divided into two categories: one uses deep learning methods to extract features, and then inputs the features into traditional machine learning classifiers such as SVM and RF for prediction; the other directly uses the deep learning method to make the final prediction. In the first category, Lv et al.^30^ proposed an ACP predictor that uses two embedding models SSA and UniRep to extract features and inputs them into 6 machine learning models such as SVM for classification, respectively. But this method is not end-to-end, and there may be some intermediate losses.

In the second category, how the sequences are represented numerically is crucial^26^. Wu et al.^31^ adopted the word2vec word embedding method to encode sequences. The Word2vec^32,33^ method learns word embedding using shallow neural networks and is widely used in natural language processing. He et al.^27^ proposed a deep learning-based predictor called ACPred-LAF, to encode sequences with a multisense-scaled embedding algorithm. These embedding methods can effectively describe the peptide sequence and retain the original information of the data, but it does not apply the prior biological information of amino acids in the numerical representation for the peptide sequences, and lacks biological interpretability to some extent. After the embedding layer, they used the encoder structure in the transformer^34^ to predict ACPs, which requires many hyperparameters to be adjusted, including the number of self-attention heads, the dimensions of query, key, and value ,etc. In 2021, Ahmed et al.^26^ developed ACP-MHCNN based on a multi-head convolutional neural network, which uses BPF, physicochemical properties of amino acids, sequence evolution information to numerically represent peptide sequences. This numerical representation method integrates multiple aspects of information and has biological significance. However, only 15 N-terminal residues of the peptide sequence are used in this method. For sequences of length greater than 15, sequence information is lost during numeralization. Given the lack of biological interpretability and loss of sequence information in existing numerical representation methods, we considered using a variety of amino acid features to numerically represent peptide sequences to make full use of the information in peptide sequences and at the same time considered the biological meaning of the numerical representation.

In this paper, we developed a new deep learning-based ACP predictor named ACPred-BMF, which is based on a peptide sequence representation method and a Bidirectional LSTM neural network framework. By integrating BPF, qualitative and quantitative properties of amino acids into one vector to numerically represent residues, we obtained a new numerical representation for peptide sequences, which contains sequence information and prior information to more comprehensively characterize the peptide sequence. Considering the structure nature of RNN recurrent connections, we used Bidirectional LSTM neural network framework to learn the order information contained in the sequences, which is difficult for traditional machine learning-based methods. We first conducted explainable prediction among deep learning-based methods for ACP prediction by using a Shapley additive explanations (SHAP) algorithm to interpret the model and obtain relatively important features for ACP prediction. Comparative experiments show that ACPred-BMF is one of the state-of-the-art predictors compared to the existing ACP prediction methods.

## Materials and methods

### Benchmark dataset

In this study, we used the benchmark datasets collected by Agrawal et al. in AntiCP2.0^23^ for model training and result comparisons. The benchmark datasets could be downloaded from https://webs.iiitd.edu.in/raghava/anticp2/download.php. It consisted of the main dataset and the alternate dataset.

(i) In the main dataset, ACPs verified in the experiment were taken as positive samples, and anti-microbial peptides (AMPs) were taken as non-ACPs, i.e., negative samples. It contains 1722 peptides of which 861 ACPs and 861 non-ACPs (or AMPs).

(ii) In the alternate dataset, ACPs and random peptides were regarded as positive samples and negative samples, respectively. It contains 1940 peptides, including 970 experimentally validated ACPs and 970 random peptides.

### Five-fold cross-validation and independent testing

K-fold cross-validation and independent testing are common methods to evaluate the quality of machine learning models. K-fold cross-validation divides the training set into K parts, and each part consisted of an equal number of positive samples and negative samples. Any K-1 parts are used for training and the other part is used as a validation set. Finally, the results of K models on their respective validation sets are averaged to obtain the K-fold cross-validation performance. The current research conducted the fivefold (*K* = 5) cross-validation.

For a fair comparison, we adopted the datasets used by Agrawal et al.^23^, in which each dataset was divided into a training dataset and an independent testing dataset in a ratio of 8:2. We conducted five-fold cross-validation on the training dataset to select the optimal parameters. Further, the model trained on the whole training dataset was used to predict the independent testing dataset, so as to obtain the performance of the model on the independent test dataset, which is the independent testing process.

### Numerical representation for peptide sequences

In the original data, the peptide sequence is a character sequence consisting of 20 amino acid characters, such as a peptide sequence $P$,

1 $$ \begin{array}{*{20}c} {P = p_{1} p_{2} p_{3} \ldots p_{L}  } \\ \end{array} $$

where $p_{i}$ represents the $i$-th residue in the peptide sequence, $i = 1,2, \ldots ,L$;${ }p_{i} \in \left\{ {A,{ }C,{ }D,{ }E,{ }F,{ }G,{ }H,{ }I,{ }K,L,M,N,P,Q,R,S,T,V,W,Y} \right\}$; $L$ represents the length of the peptide sequence $P.$ These peptide sequences vary in length, ranging from 3 to 50. The input of the deep learning model should be in the form of a numerical vector instead of a character sequence, so a numerical representation for the original data is required.

From the two perspectives of keeping the original information of the sequences as much as possible and utilizing the prior knowledge in biology, this study considered the following features to represent the sequence numerically.

#### Binary profile feature (BPF)

This feature encodes each of the 20 amino acids (A, C, D, E, F, G, H, I, K, L, M, N, P, Q, R, S, T, V, W and Y) into a 20-dimensional 0–1 vector. Specifically, A is represented as (1,0, … ,0), C is represented as (0,1, …, 0), Y is represented as (0,0, …, 1) and so on. This feature has been widely used in ACP prediction and contributes to the improvement of prediction performance^13,21,23,28^.

#### Quantitative properties of amino acids (Quanc)

Amino acids have some quantitative properties, such as molecular weight, isoelectric point, etc. However, as far as we know, the quantitative properties of amino acids rarely have been directly applied to ACP prediction. These properties can describe the differences between amino acids from multiple perspectives, and this description has practical biological significance. The quantitative properties of amino acids used here are shown in Table 1.

**Table 1:** Quantitative properties of amino acids.

Amino acid	Molecular mass^35,36^	isoelectric point^37^	pk1^38^	pk2^38^	pKa^38^	van der Waals volumes^39^
G	75.07	6.06	2.34	9.6	0	47.3
A	89.09	6.11	2.34	9.69	0	64.4
V	117.15	6.02	2.32	9.62	0	98.6
L	131.17	6.04	2.36	9.6	0	115.7
I	131.17	6.04	2.36	9.6	0	115.7
F	165.19	5.76	1.83	9.13	0	139.9
W	204.23	5.88	2.83	9.39	0	196.9
Y	181.19	5.63	2.2	9.11	10.07	136.9
D	133.1	2.98	1.88	9.6	3.65	80.1
H	155.16	7.64	1.82	9.17	6	118.9
N	132.12	5.43	2.02	8.8	0	94.6
E	147.13	3.08	2.19	9.67	4.25	97.2
K	146.19	9.47	2.18	8.95	10.53	118.1
Q	146.15	5.65	2.17	9.13	0	111.7
M	149.21	5.71	2.28	9.21	0	120.5
R	174.2	10.76	2.17	9.04	12.48	138.4
S	105.09	5.7	2.21	9.15	0	66.1
T	119.12	5.6	2.09	9.1	0	88.9
C	121.16	5.15	1.96	10.28	8.18	82.2
P	115.13	6.3	1.99	10.6	0	88

In this table, *p* $K_{a}$ = 0 is the padding data.

There are different orders of magnitude between different attributes (columns) in Table 1, and the data needs to be standardized to make properties with different measures comparable. Perform $$ z - score$$ standardization on the data in Table 1, set that the $$i$$-th row and the $$j$$-th column of the original data and standardized data in Table 1 are $$x_{ij}$$ and $$z_{ij} \left( {i = 1,2, \ldots ,20;j = 1,2, \ldots ,6} \right)$$ respectively. The calculation process of $$z_{ij}$$ is as follows:

2 $$ \begin{array}{*{20}c} {z_{ij} = \frac{{(x_{ij} - \mu_{j} ){ }}}{{\sigma_{j} }}  } \\ \end{array} $$

where

3 $$ \begin{array}{*{20}c} {\mu_{j} = \frac{{\mathop \sum \nolimits_{i = 1}^{20} x_{ij} }}{20} } \\ \end{array} $$

4 $$ \begin{array}{*{20}c} {\sigma_{j} = \sqrt {\frac{1}{20}\mathop \sum \limits_{i = 1}^{20} \left( {x_{ij} - \mu_{j} } \right)^{2} }  } \\ \end{array} $$

#### Qualitative properties of amino acids (Qualc)

Besides quantitative properties, amino acids have some qualitative physicochemical properties such as hydrophobicity, polarity, etc. In previous studies of traditional machine learning-based methods^16,18,19,21^, the qualitative physicochemical properties of amino acids have also been exploited and have been shown to help improve the performance of the predictor. We represented amino acids based on their qualitative properties ^40^, such that under a certain property (such as charged), amino acids with the same class have the same representation. Based on this consideration, the qualitative properties of amino acids (Qualc) are proposed in this paper, which are shown in Table 2.

**Table 2:** Qualitative properties of amino acids.

Qualitative Properties	Category	Amino acid
hydrophobicity^40^	Y	G, A, V, L, I, F, W, Y, H, K, M, T, C
	N	D, N, E, Q, R, S, P
polarity^40^	Y	W, Y, D, H, N, E, K, Q, R, S, T, C
	N	G, A, V, L, I, F, M, P
charge^40^	Negative	D, E
	Positive	H, K, R
	N	G, A, V, L, I, F, W, Y, N, Q, M, S, T, C, P
Aromatic or aliphatic^41^	Aromatic	F, W, Y, H
	Aliphatic	L, I, V
	N	G, A, D, N, E, K, Q, M, R, S, T, C, P

In this table, ‘Y’ and ‘N’ indicate amino acid categories with and without the corresponding property (in the first column).

Since the physicochemical properties of the amino acids described in Table 2 are in the form of characters, they cannot be directly used to represent the peptide sequence numerically. So, the raw data in Table 2 needs to be one-hot encoded to convert character data to numeric data, i.e. set $$n $$ variables for the $$n$$ categories of each property, if the amino acid belongs to the category corresponding to the variable under this property, the variable value is 1, otherwise, it is 0. For example, for charge, set three variables "charge_positive", “charge_negative”, "charge_N", for the amino acid $$p_{i}$$, there are:

5 $$ \begin{array}{*{20}c} {charge\_positive\left( {p_{i} } \right) = \left\{ {\begin{array}{*{20}c} {1,p_{i} is positive} \\ {0,p_{i} is not positive} \\ \end{array} } \right. } \\ \end{array} $$

6 $$ \begin{array}{*{20}c} {charge\_negative\left( {p_{i} } \right) = \left\{ {\begin{array}{*{20}c} {1,p_{i} is negative} \\ {0,p_{i} is not negative} \\ \end{array} } \right. } \\ \end{array} $$

7 $$ \begin{array}{*{20}c} {charge\_N\left( {p_{i} } \right) = \left\{ {\begin{array}{*{20}c} {1,p_{i} is not charged} \\ {0,p_{i} is charged} \\ \end{array} } \right. } \\ \end{array} $$

#### Feature combinations

The three features proposed above describe 20 amino acids from different perspectives, and we considered the use of feature combinations for numerical representation. Since each vector encoded by BPF has a one-to-one correspondence with amino acids, which is an essential feature, BPF representation is used in each feature combination. We considered the 4 feature combinations listed in Table 3.

**Table 3:** Feature combinations and their coding dimensions.

Feature combination	Coding dimension of single residue
BPF(bpf)	20
BPF + Quanc (quanc)	26
BPF + Qualc (quanl)	30
BPF + Quanc + Qualc (mix)	36

In the first column, the content in parentheses represents the abbreviation of the feature combination.

Let the numerical representation dimension of each residue be $d$ ($d$ may be 20, 26, 30 or 36). For a sequence of length $l$, encode it into a $L_{max} \times d$-dimensional matrix: the first $l$ rows are the numerical representation of the peptide sequence; for the $\left( {l + 1} \right)$-th row to $L_{max}$-th row, the same value is used for the padding operation. $L_{{{\text{max}}}}$ is the longest sequence length in the data, in this paper $L_{{{\text{max}}}} = 50$.

### Deep learning methods

#### Recurrent neural network (RNN)

The core idea of the recurrent neural network^41,42^ is to transfer the historical information to the current moment, and use it together with the input of the current moment to generate the output of the current moment. In this way, the model has a memory along time, that is, it can retain the order information of the data, which is suitable for processing sequence data such as peptide sequences. The recurrent neural network updates the hidden state at time $t$ by the Eq. (8):

8 $$ \begin{array}{*{20}c} {h_{t} = f\left( {h_{t - 1} ,x_{t} } \right) } \\ \end{array} $$

where $h_{0} = 0$; $h_{t}$ represents the hidden state at time $t$; $x_{t}$ represents the input at time $t$; $f\left( \cdot \right)$ is a nonlinear function.

In actual operation, the performance of RNN is not ideal, especially when dealing with long sequences. Hochreiter^43^ theoretically explained the reason why RNN is difficult to deal with long-distance dependencies and innovatively proposed a new network architecture Long-Short Term Memory(LSTM) to remedy it. As a variant of RNN, LSTM^43,44^ often works better in experiments, so we used the LSTM model.

#### Bidirectional recurrent neural network

Schuster et al.^45^ proposed a bidirectional RNN, that is, a hidden layer is established through the original sequence, and then a hidden layer is established with the original sequence in reverse order, and the generated two hidden layer sequences are aggregated to obtain the final output result. This bidirectional idea is easy to generalize to variants of RNNs, such as Bidirectional LSTM (Bi-LSTM), Bidirectional GRU^46^, etc. We used a Bi-LSTM based on the following considerations: For a residue in a peptide sequence, not only the following residues have an effect on it, but also the preceding residues.

#### Attention mechanism

Attention is a complex cognitive function that is essential for humans^47–49^. When visually perceiving things, humans typically do not see the entire scene from start to finish, but instead observe and focus on specific parts and ignore others^49^. Based on this visual mechanism, the attention mechanism was proposed.

##### Attention layer

In a peptide sequence, the information contained in it is not equally important. Gautam et al.^12^ demonstrated that there may be specific amino acids at the N-terminal and C-terminal positions of the peptide sequence. In order to focus the model on the N- and C-terminal information of the peptide sequences, we used the attention mechanism. For the concrete method, we referred to the attention mechanism realization of Zhou et al.^50^, which was applied in the task of relation classification.

Suppose the output of the $i$-th sequence passes through a Self-attention layer is $h_{i}$, the trainable parameters $W \in {\mathbb{R}}^{2n} , b \in {\mathbb{R}}^{{L_{max} }}$. The mathematical description of attention is as follows:

9 $$ \begin{array}{*{20}c} {e_{i} = h_{i} W + b } \\ \end{array} $$

10 $$ \begin{array}{*{20}c} {\alpha_{ij} = \frac{{\exp \left( {e_{ij} } \right)}}{{\mathop \sum \nolimits_{k} \exp \left( {e_{ik} } \right) }},j = 1,2, \ldots ,L_{max}  } \\ \end{array} $$

11 $$ \begin{array}{*{20}c} {att\left( {h_{i} } \right) = \mathop \sum \limits_{j = 1}^{{L_{max} }} \alpha_{ij} h_{ij} . } \\ \end{array} $$

where $$h_{i} \in {\mathbb{R}}^{{L_{{{\text{max}}}} \times 2n}}$$, $$e_{i} \in {\mathbb{R}}^{{L_{max} }}$$. Let the $$j$$-th row of $$h_{i}$$ and the $$j$$-th element of $$e_{i}$$ be $$ h_{ij}$$ and $$e_{ij}$$ respectively. Then for the $$i$$-th sequence, the relative importance $$\alpha_{ij}$$ of the $$j$$-th residue is $$\alpha_{ij}$$. $$att\left( {h_{i} } \right)$$ is the weighted sum of each row of $$h_{i}$$ (information for each residue). $$n $$ is the number of units in the Bi-LSTM layer.

##### Self-attention mechanism

Self-attention, is an attention mechanism relating different positions of a single sequence in order to compute a representation of the sequence^34^. Using the self-attention mechanism, the relationship between any two residues in a peptide sequence can be directly established, regardless of the distance between them. Two residues that are far apart in the sequence may be relatively close in space and to some extent have an internal connection, so we considered using self-attention mechanism to establish the relationship between any two residues. The self-attention mechanism can be implemented using the "SeqSelfAttention" function in Keras, and its mathematical description is as follows:

12 $$ \begin{array}{*{20}c} {h_{{t,t^{\prime}}} = \tanh \left( {x_{t}^{T} W_{t} + x_{{t^{\prime}}}^{T} W_{x} + b_{t} } \right) } \\ \end{array} $$

13 $$ \begin{array}{*{20}c} {e_{{t,t^{\prime}}} = \sigma \left( {W_{a} h_{{t,t^{\prime}}} + b_{a} } \right)} \\ \end{array} $$

14 $$ \begin{array}{*{20}c} {a_{t} = softmax\left( {e_{t} } \right) } \\ \end{array} $$

15 $$ \begin{array}{*{20}c} {l_{t} = \mathop \sum \limits_{{t^{\prime}}} a_{{t,t^{\prime}}} x_{{t^{\prime}}}  } \\ \end{array} $$

where $a_{t} \in {\mathbb{R}}^{{L_{max} }}$, $x_{t} \in {\mathbb{R}}^{{ 2n}}$ represents the output of the Bi-LSTM layer at time $t$ and the value $a_{{t,t^{\prime}}}$ represents the relative importance of the input at the $t^{\prime}$-th position to the input at the $t$-th position.

### Model overview

#### BMF-basic

**Figure 1:**
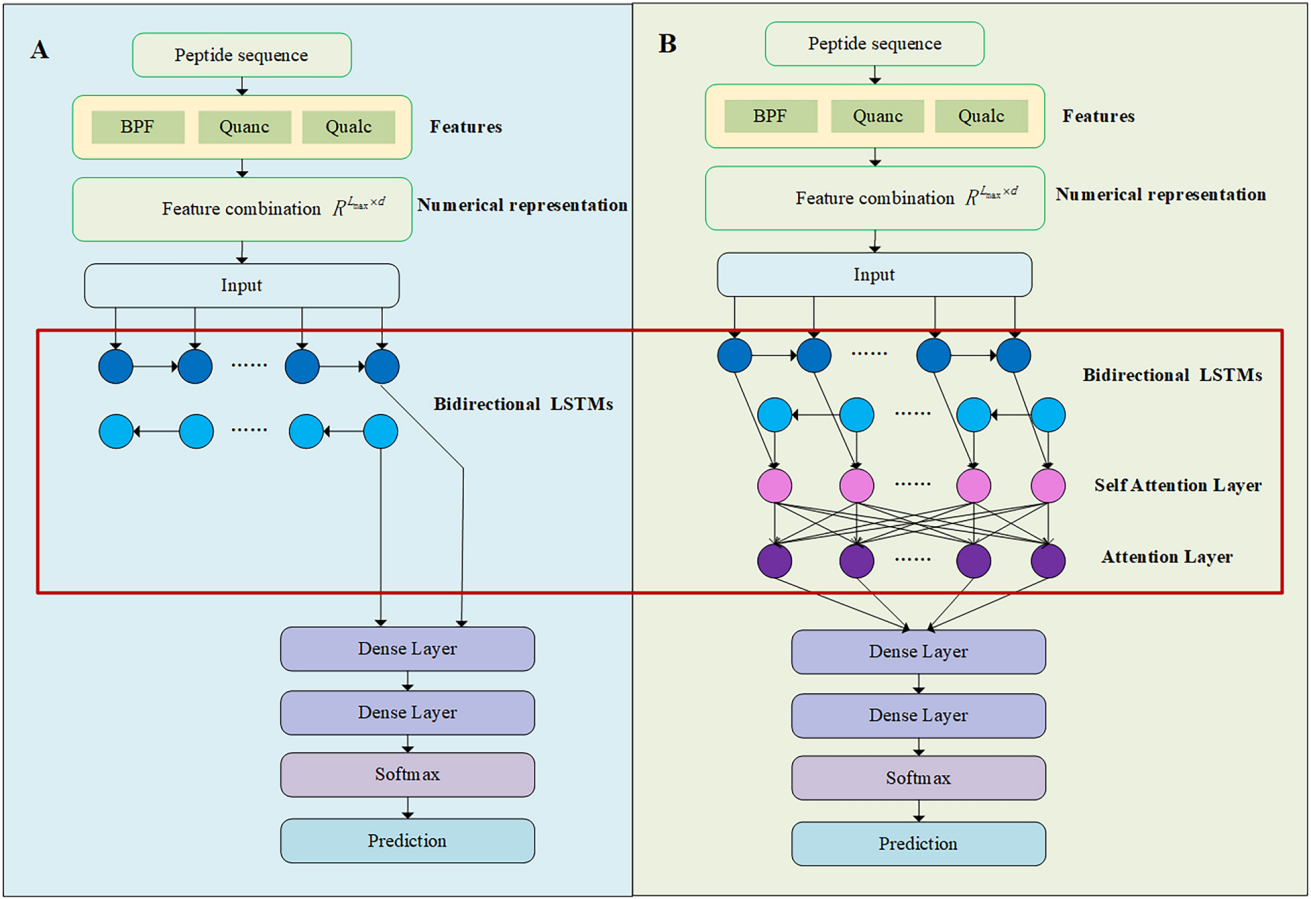
The framework of the proposed ACPred-BMF with multi feature representations. We proposed two models: (**A**)BMF-Basic and (**B**)BMF-Selfatt. For BMF-Basic and BMF-Selfatt, we all used the feature combination of BPF, Quanc and Qualc to numerical representation for peptide sequences. The red box marks the difference between the two models: whether to include the attention layers or not.

Firstly, consider the use of a Bi-LSTM model: the first layer is a Bidirectional LSTM layer; the second layer is a fully connected layer, which further extracts and synthesizes the output of the LSTM layer; the last layer is a fully connected layer whose activation function is set to softmax. The output of the final layer is the probability of being predicted to belong to ACPs or non-ACPs. The flow chart is shown in Fig. 1A. The Bidirectional LSTM model is a relatively basic architecture, which we call it BMF-Basic.

#### BMF-selfatt

We further considered adding attention mechanism, including ordinary attention and self-attention. We added the self-attention layer after the Bi-LSTM layer to establish the relationship between any two residues and the Attention layer after the self-attention layer to calculate the weighted sum of residue information at different positions. Same as BMF-Basic, the last two layers of the model are fully connected layers. The schematic diagram of the model is shown in Fig. 1B. Compared with BMF-Basic, this method is more complex, has more model parameters, and is more refined in the processing of sequences. We call it BMF-Selfatt.

### Evaluation metrics and methods

To comprehensively evaluate the model performance, this study has taken into account Accuracy (ACC), sensitivity (Sen), specificity (Spc), Matthew’s correlation coefficient (MCC) and area under ROC curve (AUC). These evaluation metrics are defined in the following equations:

16 $$ \begin{array}{*{20}c} {ACC = \frac{TP + TN}{{TP + TN + FP + FN}} \times 100\% } \\ \end{array} $$

17 $$ \begin{array}{*{20}c} {Sen = \frac{TP}{{TP + FN}} \times 100\% } \\ \end{array} $$

18 $$ \begin{array}{*{20}c} {Spc = \frac{TN}{{TN + FP}} \times 100\%  } \\ \end{array} $$

19 $$ \begin{array}{*{20}c} {MCC = \frac{TP \times TN - FP \times FN}{{\sqrt {\left( {TP + FP} \right) \times \left( {TP + FN} \right) \times \left( {TN + FP} \right) \times \left( {TN + FN} \right)} }} } \\ \end{array} $$

where TP, TN, FP and FN represent true positive, true negative, false positive and false negative, respectively. Corresponding to the concrete problem of ACP prediction: TP is the number of ACPs that are correctly predicted; TN is the number of non-ACPs that are correctly predicted. FP is the number of non-ACPs that are predicted as ACPs; FN is the number of ACPs that are predicted as non-ACPs.

MCC is an overall performance evaluation metric of the quality of binary classification, which returns a value between − 1 and + 1. The higher the MCC value achieves, the better the performance of the prediction model is. AUC is defined as the area enclosed by the coordinate axis and the ROC curve, which returns a value between 0 and 1. When the AUC value closes to 1, the prediction model is regarded as a better one. Among them, MCC is a very stringent metric by taking into account both accuracy and error rates of the two classes^51^. Therefore, we regarded MCC as the most important metric for feature selection, model selection and model optimization.

## Results

### Initial results

To select better numerical representations for peptide sequences and deep learning models, we used the two frameworks (BMF-Basic, BMF-Selfatt) to develop 2 models for each feature combination, a total of 8 models have been developed. We conducted experiments on both the main and alternate datasets, selected features and models through five-fold cross-validation results.

#### Initial performance of models trained on the main dataset

For the main dataset, with epochs fixed to 65, the results are shown in Table 4. It shows that under the premise of using the same feature combination, BMF-Basic has better cross-validation scores (MCC, ACC, AUC) than BMF-Selfatt. The BMF-Basic based on the BPF + Quanc + Qualc feature combination (mix) achieved the best cross-validation MCC (0.497) and ACC (74.75%) as compared with other combination of features and models. In addition, the BMF-Selfatt model using feature combinations of more than just BPF (quanc, qualc, mix) all have better cross-validation scores than the BMF-Selfatt model only using BPF except for Spc. For the BMF-Basic model, the BPF + Quanc + Qualc feature combination (mix) also has a higher cross-validation scores than the bpf except Sen. This shows that the addition of prior information of amino acids on the basis of BPF representation is helpful for ACP prediction, which may be due to the more comprehensive characterization of peptide sequences.

**Table 4:** The initial five-fold cross-validation results of models developed on main dataset.

Model	Feature combination	ACC (%)	Sen (%)	Spc (%)	MCC	AUC
Basic	bpf	74.60	76.05	73.15	0.493	0.823
	quanc	74.24	72.56	75.92	0.488	0.822
	qualc	73.44	72.86	74.03	0.470	0.815
	mix	**74.75**	**73.72**	**75.77**	**0.497**	**0.825**
Selfatt	bpf	72.28	70.10	74.45	0.449	0.801
	quanc	**73.37**	**74.16**	**72.58**	**0.469**	**0.802**
	qualc	73.00	75.60	70.39	0.463	0.812
	mix	72.64	71.83	73.44	0.456	0.820

The BMF-Basic and BMF-Selfatt initial results with the best five-fold cross-validation MCC are marked in bold.

#### Initial performance of models trained on the alternate dataset

The same experiment was also performed on the alternate dataset, with epochs fixed to 35, and the cross-validation results are shown in Table 5. It shows that whether it is BMF-Basic or BMF-Selfatt, using the BPF + Quanc + Qualc feature combination (mix) has better five-fold cross-validation scores except for Spc or Sen. Among them, BMF-Basic five-fold cross-validation scores of five metrics (MCC = 0.826, ACC = 91.24%, Sen = 91.75%, Spc = 90.72%, AUC = 0.965) were all better than the BMF-Selfatt (MCC = 0.781, ACC = 88.92%, Sen = 87.76%, Spc = 90.07%, AUC = 0.961).It can also be seen from the results that on the basis of BPF, with the same model the sequence representation using the physicochemical properties of amino acids achieves better five-fold cross-validation MCC, ACC, and AUC except for Selfatt model based on qualc.

**Table 5:** The initial five-fold cross-validation results of models developed on alternate dataset.

Model	Feature combination	ACC (%)	Sen (%)	Spc (%)	MCC	AUC
Basic	bpf	88.40	88.79	88.01	0.770	0.949
	quanc	90.34	89.82	90.85	0.808	0.961
	qualc	89.24	90.85	87.62	0.786	0.957
	mix	**91.24**	**91.75**	**90.72**	**0.826**	**0.965**
Selfatt	bpf	88.47	88.14	88.78	0.771	0.951
	quanc	88.60	90.46	86.72	0.775	0.956
	qualc	87.24	89.30	85.18	0.748	0.948
	mix	**88.92**	**87.76**	**90.07**	**0.781**	**0.961**

The BMF-Basic and BMF-Selfatt initial results with the best five-fold cross-validation MCC are marked in bold.

#### Comparison of initial results of models

In the initial results of the model, we first noticed that the use of prior information such as quantitative and qualitative properties of amino acids on the basis of BPF can effectively represent peptide sequences.

Secondly, it can be observed from the results in Tables 4 and 5 that the BMF-Basic model performs better five-fold cross-validation results than BMF-Selfatt model whether it is developed on the main dataset or the alternate dataset.

Additionally, the results show that the effective feature combination are the same in the two datasets, whose experimental results are shown in Table 6. Whether in the main dataset or alternate dataset, using the BPF + Quanc + Qualc feature combination (mix) to numerically represent the peptide sequence works best.

**Table 6:** Comparison of initial cross-validation results of the models developed on the main dataset and alternate dataset.

Dataset	Model	Feature combination	ACC (%)	Sen (%)	Spc (%)	MCC	AUC
Main	Basic	mix	74.75	73.72	75.77	0.497	0.825
Alternate	Basic	mix	91.24	91.75	90.72	0.826	0.965

### Comparison of numerical representation and embedding methods

We also made a comparison and selection between our proposed numerical representation method and the embedding method through five-fold cross-validation results. Embedding methods utilize peptide sequences information to numerically represent peptide sequences without using known prior information. Specifically, for each amino acid, it is randomly initialized into a $d_{0}$-dimensional vector. Further through model training, the $d_{0}$-dimensional vector is adaptively adjusted^27^. For a fair comparison, we set embedding and the numerical representation to have the same dimension, i.e. $d_{0} = 36$. Other model parameters are the same as numerical representation method, and the cross-validation results are shown in Table 7. On the main dataset, our representation method achieves better five-fold cross-validation scores (MCC = 0.497, ACC = 74.75%) than embedding method (MCC = 0.477, ACC = 73.73%). On the alternate dataset, the five-fold cross-validation scores of 5 metrics achieved by our representation method are all better than the embedding method. And because our representation has biological significance, while the specific meaning of each dimension of the vector obtained by the embedding method is unknown, as shown in the “Explainable prediction” section, our representation method is more interpretable than the embedding method. The results show that our proposed representation has advantages to some extent compared with the embedding method, indicating that using some prior information is beneficial to ACP prediction.

**Table 7:** Five-fold cross-validation results of the numerical representation and embedding method.

Dataset	Model	Feature combination	ACC (%)	Sen (%)	Spc (%)	MCC	AUC
Main	Basic	mix	74.75	73.72	75.77	0.497	0.825
		Embedding	73.73	74.75	72.72	0.477	0.815
Alternate		mix	91.24	91.75	90.72	0.826	0.965
		Embedding	87.95	89.04	86.85	0.761	0.946

### Model optimization

According to the above results, we optimized the model (the BMF-Basic with the BPF + Quanc + Qualc feature combination) which achieves the best initial results. We used the hyper-parameter grid search method to determine the optimal model based on the main and alternate datasets.

#### Main dataset model optimization

**Figure 2:**
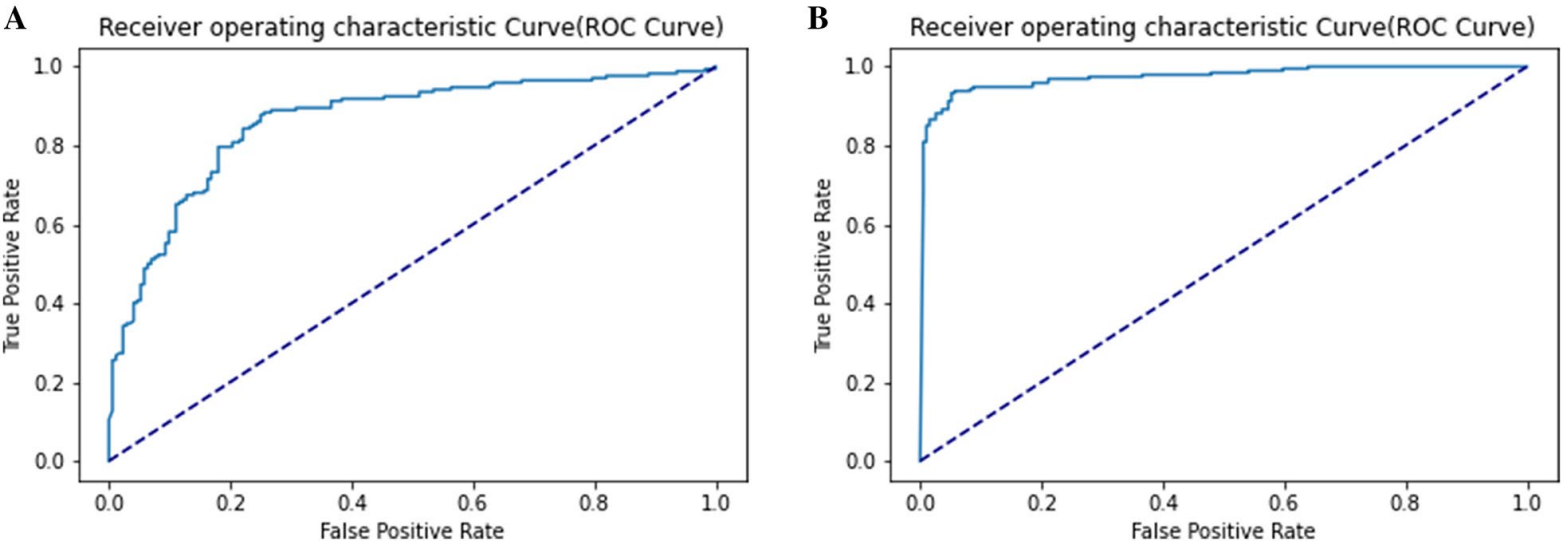
ROC curve of the optimized model over independent test set. (**A**)The ROC curve of the optimized model on the main dataset. (**B**)The ROC curve of the optimized model on the alternate dataset.

The experimental results of the five-fold cross-validation are shown in Supplementary data S1. It can be observed that when the number of units in the Bi-LSTM layer is 64, the number of neurons in the first fully connected layer is 50, and the number of epochs is 45, the model has the best cross-validation MCC (0.516). Table 8 shows the five-fold cross-validation and independent test results corresponding to the optimal model. On the main dataset, the model realized the independent test results of MCC = 0.623 and ACC = 80.81%. And the ROC curve for optimized model based on main dataset is shown in Fig. 2A.

**Table 8:** The performance of optimized models based on the main dataset and alternate dataset.

Dataset	Five-fold cross-validation					Independent test				
	ACC (%)	Sen (%)	Spc (%)	MCC	AUC	ACC (%)	Sen (%)	Spc (%)	MCC	AUC
Main	75.76	76.93	74.60	0.516	0.827	80.81	88.37	73.26	0.623	0.861
Alternate	91.49	90.85	92.14	0.831	0.968	93.56	92.27	94.85	0.871	0.974

The optimized model uses BMF-Basic with BPF + Quanc + Qualc feature combination (mix).

#### Analysis of optimization results

The experimental results of the five-fold cross-validation are shown in Supplementary data S2. On the alternate dataset, when the number of units in the Bi-LSTM is 128, the number of neurons in the first fully connected layer is 50, and the number of epochs is 35, there is the best five-fold cross-validation MCC (0.831). The five-fold cross-validation and independent test results of the optimized model are shown in Table 8. The optimized model performed AUC of 0.974 on the test dataset, as shown in Fig. 2B.

After model optimization, the model applied on the main dataset has a higher number of epochs, while the model on the alternate dataset has fewer epochs. After our analysis, we got a similar view to He et al.^27^, which may be due to the difference in the task difficulty itself between the two datasets: on the main dataset, ACPs and AMPs need to be distinguished; On the alternate dataset, ACPs need to be distinguished from random peptides. Relatively speaking, the range of AMPs is smaller, the discrimination between ACPs and AMPs is smaller, for ACPs are part of the AMP group^23^ and the task is more difficult, requiring more epochs to train. Distinguishing ACPs from random peptides is relatively simple, and the model requires fewer epochs.

In addition, we also tested models on the non-redundant test sets, in which sequences similar to training sets were removed. The original test datasets all have sequences similar to the training sets. For example, the main training data includes the sequence “GLFDIVKKVVGTIAGL”, and the test data includes a similar sequence “GLFDIVKKVVGTLAGL”. In order to obtain more objective results, we used CD-HIT-2D in the CD-HIT program^52^ to compare the training set with the test set, and retain the test set sequence that is not similar to the training set sequence (below the sequence identity threshold) for independent testing using the optimized models. According to the facts that if a protein sequence has 40% or more similarity to another with a known function, it is highly probable that both perform the same function^53^ and at the same time according to the thresholds used in the references, we conducted experiments on three thresholds: 40%^53^, 80%^20^ and 90%^19,54^ (Table 9).

**Table 9:** Independent test results on non-redundant datasets (sequence identity threshold: 100%, 90%, 80%, 40%).

Threshold	Main dataset				Alternate dataset			
	ACC (%)	Sen (%)	Spc (%)	MCC	ACC (%)	Sen (%)	Spc (%)	MCC
100%	80.81	88.37	73.26	0.62	93.56	92.27	94.85	0.87
90%	78.54	84.31	74.05	0.58	92.38	88.43	94.85	0.84
80%	75.90	80.56	72.34	0.52	92.03	85.54	94.82	0.81
40%	72.73	46.15	90.00	0.41	95.24	86.36	96.80	0.82

After removing redundancy, the independent test MCC of the two models all decrease. When the threshold value is set at 90%, the independent testing MCC and ACC on the main dataset are 0.04 and 2.27% lower than the original independent testing results; compared with the original independent test results, the independent testing MCC and ACC on the alternate dataset decrease by 0.03 and 1.18%. When the threshold is 90%, the independent testing scores decrease slightly. The big drop in independent test scores is the model based on the main dataset, when tested on the test set with 40% threshold. In addition to the performance of the model itself, it may also be related to the small amount of data (only 33 sequences remain after the main test set is de redundant with 40% threshold), which may not be representative. When the threshold value is 40%, the independent test scores obtained on the alternate dataset not change much compared with the original independent test scores: MCC decreases by 0.05 and ACC increases by 1.68%. It shows that our model has good prediction performance on non-redundant test sets, and has generalization ability.

### Network feature visualization

**Figure 3:**
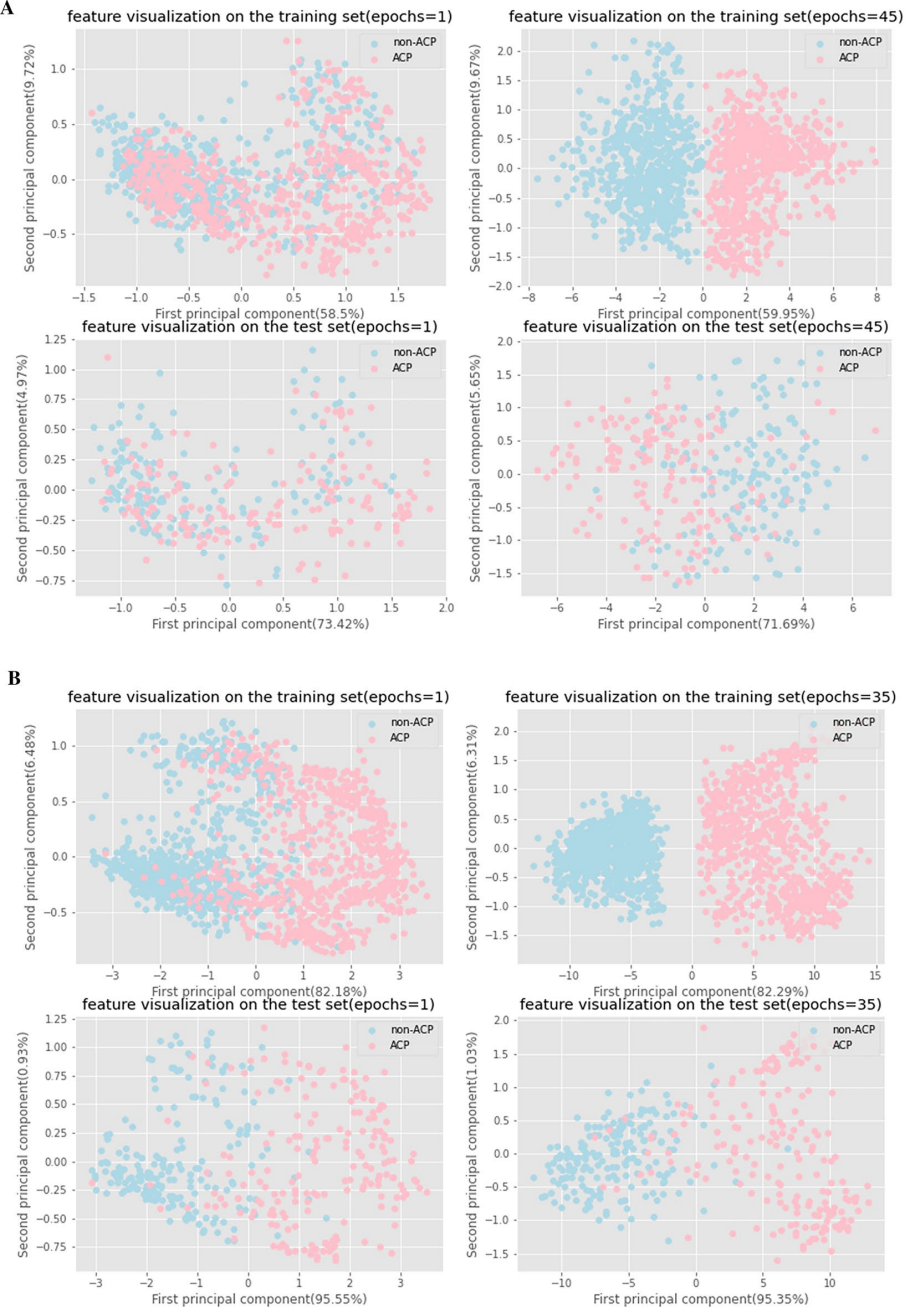
Feature visualization by PCA for dimension reduction. (**A**) Dimension reduction of each sample on the main dataset. (**B**) Dimension reduction of each sample on the alternate dataset. The horizontal axis represents the first principal component, and the vertical axis represents the second principal component. The numbers in brackets of the horizontal and vertical axis labels indicate the explained variance ratio of the corresponding principal components. Pink points represent ACPs, while light blue points represent non-ACPs.

Deep learning-based methods can automatically extract features^29^. To demonstrate the effectiveness of the model in extracting features, we further visualized the optimized model. Specifically, we output the result of the penultimate layer of the network (that is, the first fully connected layer) to obtain a high-dimensional feature. High-dimensional features cannot be directly visualized. Principal component analysis (PCA)^55^ obtains a new variable, the principal component, by linearly transforming the original variable, and maximizes the variance of the principal component to contain more information. In this way, most information can be concentrated in the previous principal components, and the use of principal component analysis can effectively reduce dimensionality. We use PCA^55^ dimensionality reduction technique to reduce our obtained high-dimensional features to 2 dimensions to facilitate visualization. Figure 3A and B show the discriminatory effect of automatically extracted features on the main dataset and the alternate dataset, respectively. At the beginning (epochs = 1), the points representing ACPs and non-ACPs are mixed together because the parameters of the neural network are randomly initialized. After training (epochs = 35/45), the ACPs and non-ACPs in the training set can be well distinguished by the features automatically extracted by the optimized models. The extracted features can effectively distinguish ACPs from non-ACPs in the test set even if the network does not use the data in the test set during training. This shows that the models we trained have learned some common features, not just the features that distinguish ACPs from non-ACPs on the training set, and the models are generalizable to some extent.

### Explainable predictions

At present, the prediction method based on deep learning has promoted the development of ACP prediction, but deep learning has the underlying black-box nature, which is reflected in that it is difficult to know its prediction mechanism and further explore after getting the results of the model. It arises from the fact that, despite having the underlying statistical principles, there is a lack of ability to explicitly represent the knowledge for a given task performed by a deep neural network^56^. Intelligibility means that the model is easily understandable^57^. The explicability of the model is important for ACP prediction, it can provide an explanation on the underlying mechanism of the biological activity of ACPs, which is more useful to further analyze the characteristics of the anticancer activity of peptides^58^, thus promoting the discovery of more ACPs. Secondly, explicability can enhance the reliability of the model. A machine learning algorithm should be considered reliable in the way it allows to extract more knowledge and information than just having a prediction at hand^59^. Thirdly, explicability is the key to trust-able use of the deep learning model and a key enabler for its deployment in the real world^56^. By showing how the model makes decisions can inculcate trust among the end-users^56^. Last but not least, considering that the model will be extended to peptides with different biological activities in the future, the main features of peptides with different functions can be identified by utilizing the explicability, thus promoting the development of peptide drugs.

Based on the above considerations, we further analyzed the results using SHAP (Sharply Additive exPlanations)^60^ to interpret our model and alleviate the black-box prediction problem in deep learning, which is a generalized metric for feature importance and utilizes the game-theory-based Shapely value to calculate the contribution of each feature to the model’s output^61^. The SHAP formula is:

$$ \begin{array}{*{20}c} {g\left( {z^{\prime}} \right) = \phi_{0} + \mathop \sum \limits_{i = 1}^{M} \phi_{i} z_{i} ^{\prime} \left( {20} \right)} \\ \end{array} $$

where $g$ is the explanatory model, which approximates the output of the original model; $M$ is the number of input features; $z^{\prime} \in \left\{ {0,1} \right\}^{M}$ indicates whether the corresponding feature exists; $\phi_{i}$ is the attribution value of each feature; $\phi_{0}$ is a constant. That is, the sum of the Shapley values for all features plus the mean prediction equals the actual prediction^62^. This is not the same as direct feature effects known from (generalised) linear models, and the SHAP value for a feature should be seen as its compound effect when interacting with the other features. We obtained the SHAP value through the shap package in Python.

We mainly analyzed the model developed on the alternate dataset for that in the alternate dataset, using random peptides as negative samples, as they are more distinguishable from ACPs and can highlight the main features of ACPs.

**Figure 4:**
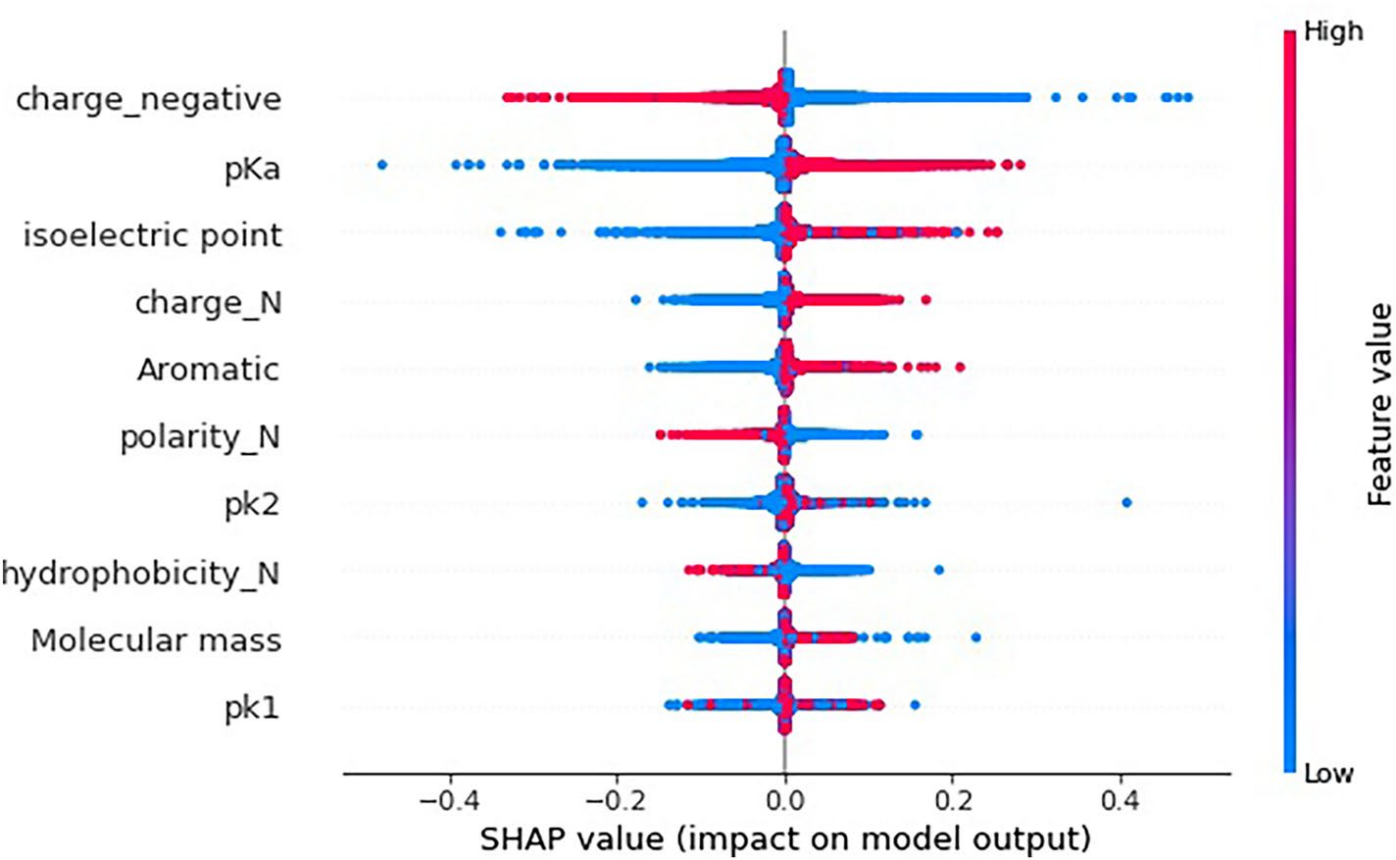
The impact of the features on ACP prediction. The $x$-axis of the figure labeled as the SHAP value (impact on model output), and the $y$-axis, listing the top 10 features, are ranked based on their contribution to the ACP prediction. The red color corresponds to high value of the features consisting of Quanc and Qualc, whereas the blue color corresponds to low value of the features. The naming of features is consistent with the aforementioned numerical representation, such as "charge_N" means a feature without charge (neutral charge feature).

**Figure 5:**
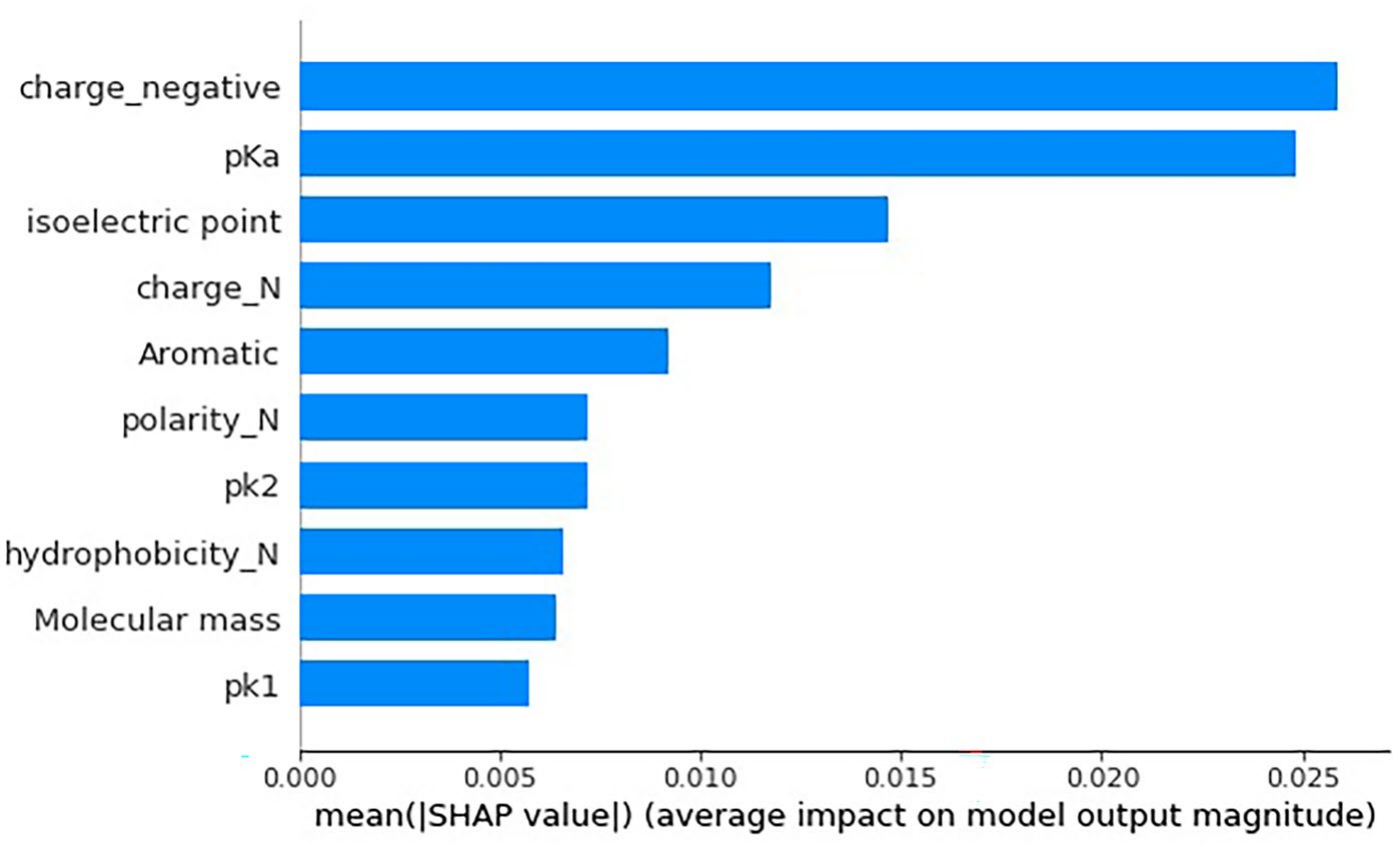
Feature importance rank using the SHAP method. The figure shows the top ten features with the highest importance, where the longer the bars’ length is, the more important or the more contribution of the feature for ACP prediction.

Figure 4 and 5 show the top 10 Quanc, Qualc features ranked using SHAP. Each point in Fig. 4 represents the impact of a feature at a position on the ACP prediction for a peptide sequence. We also calculated the feature importance: take the average value of the SHAP values’ absolute values of each feature as the importance of the feature, and get a bar chart (as shown in Fig. 5). Figure 4 shows that negatively charge is the first-ranked importance factor impacting ACP prediction, and lower values of this feature result in higher SHAP values, which correspond to a higher probability that a peptide sequence be an ACP. That is, negatively charged amino acids have a negative impact on the probability of being predicted to be ACPs, and positively or neutral amino acids have a positive impact. In addition, aromaticity is also an important feature, ranking fifth, and Fig. 4 shows that aromatic amino acids have a positive impact on ACP prediction. The above analysis is consistent with existing studies: Agrawal et al.^23^ pointed out that ACPs are rich in positively charged residues and aromatic amino acids. The second most important feature is p $$K_{a} $$(acidity coefficient), which with high value has a positive impact on ACP prediction. Existing studies have also shown that the theoretical interpretation and prediction of protein p $$K_{a}$$ value is helpful for understanding many biochemical problems^63^. Similarly, points with higher values under the isoelectric point (the pH value when a molecule has no charge on the surface) or neutral charge feature are on the positive side of SHAP value, indicating that the higher values of these two features also correspond to a positive impact on ACP prediction. Through the SHAP algorithm, we obtained the feature importance rank based on their contribution to ACP prediction and the influence direction (positive or negative correlation) on the predicted probability of being ACPs, which is consistent with the existing research results and helps to further understand the mechanism of ACPs.

### Comparison with the existing methods

We also compared our ACPred-BMF predictor to the existing methods on independent test sets. The independent testing results for state-of-the-art ACP prediction directly came from references^23,26,27,58^. The results are shown in Table 10. In the main dataset, the MCC and accuracy (0.62, 80.81%) of our method are only lower than iACP-FSCM, whose MCC and accuracy are 0.65, 82.5%. The Spc (73.26%) of ACPred-BMF ranked fifth, but its Sen (88.37%) ranked second. Only the Sen (100%) of the AntiCP is higher than ACPred- BMF, but the MCC of the AntiCP is only 0.07. ACPred-BMF has both high Sen and ACC, so it is less likely to miss the real ACPs on the premise of high overall accuracy. In the alternate dataset, MCC and the accuracy (0.87, 93.56%) of our method ACPred-BMF achieve the best prediction performance compared with other methods. ACPred-BMF discriminates ACPs and non-ACPs with balanced Sen (92.27%) and Spc (94.85%).

**Table 10:** Comparison of the independent testing metrics values for ACPred-BMF with state-of-the-art ACP predictors on the main and alternate datasets.

Methods	Main dataset				Alternate dataset			
	ACC (%)	Sen (%)	Spc (%)	MCC	ACC (%)	Sen (%)	Spc (%)	MCC
ACPred-BMF	80.81	88.37	73.26	0.62	93.56	92.27	94.85	0.87
AntiCP-2.0	75.43	77.46	73.41	0.51	92.01	92.27	91.75	0.84
AntiCP	50.58	100.00	1.16	0.07	89.95	89.69	90.20	0.80
ACPred	53.47	85.55	21.39	0.09	85.31	87.11	83.51	0.71
ACPred-FL	44.80	67.05	22.54	− 0.12	43.80	60.21	25.58	− 0.15
ACPpred-Fuse	68.90	69.19	68.60	0.38	78.87	64.43	93.30	0.60
PEPred-Suite	53.49	33.14	73.84	0.08	57.47	40.21	74.74	0.16
iACP	55.10	77.91	32.16	0.11	77.58	78.35	76.80	0.55
iACPred-FSCM	82.50	72.60	90.30	0.65	88.90	87.60	90.20	0.78
ACPred-LAF	79.07	81.98	76.16	0.58	93.30	93.30	93.30	0.87
ACP-MHCNN	73.00	78.50	67.40	0.46	90.00	86.60	86.60	0.81

Although iACP-FSCM has a better independent test MCC, Spc and ACC on the main dataset than ACPred-BMF, ACPred-BMF has better test scores (MCC, ACC, Sen, Spc) than iACP-FSCM on the alternate dataset. Considering that more data are used and more kinds of peptides are involved in the future, our proposed deep learning based-model is more adaptable: deep learning is data-driven, highly dependent on data, and in general, the larger the amount of data, the better its performance within limits; deep learning has strong adaptability and can learn very complex functions with the composition of enough such transformations^29^. Compared with other models except for iACP-FSCM, ACPred-BMF has better independent test performance and explainability, and can give important features for prediction.

The observed results show that our method, ACPred-BMF, is one of the state-of-the-art predictors based on machine learning and deep learning methods.

## Conclusion

Compared with traditional treatment methods, ACPs have great therapeutic potential. However, experimentally identifying ACPs is time-consuming, laborious and expensive. We investigated this problem and proposed a predictor called ACPred-BMF that uses the Bi-LSTM network and a new numerical representation for peptide sequences. In terms of the numerical representation for peptide sequences, we characterized peptide sequences from the perspectives of using prior biological knowledge and retaining original information. We used BPF, quantitative and qualitative properties of amino acids and their combinations to represent peptide sequences. For the model, we considered two network architectures without and with attention: BMF-Basic, BMF-Selfatt. The results show that the BMF-Basic performs better five-fold cross-validation effects for the experimental data in this paper. We also visualized the features automatically extracted by the network, showing that the feature can well distinguish ACPs from non-ACPs. Using the SHAP technique, we further interpreted the model and found that features such as charge, p $K_{a}$, and aromaticity play an important role in predicting ACPs.

In future work, we will use more complex models such as transformers^34^ to adapt more complex problems, such as multifunctional classification of peptides. In addition, it can also be considered to assign different weights to features and residues at different positions through the attention mechanism to obtain a weighted numerical representation of the peptide sequences. By definition, a counterfactual is the smallest variation of the input such that it changes the predicted behaviour^59^. Furthermore, we can consider combining counterfactual theory to design ACP, that is, by appropriately modifying the sequence of non-ACP to make it have anti-cancer activity.

Experimental results show that our proposed predictor, ACPred-BMF, is quite competitive with existing prediction methods and is one of the state-of-the-art ACP predictors. Our study provides new ideas for the prediction of ACPs, especially on deep learning-based methods for ACP prediction. Besides, a web server as the implementation of ACPred-BMF can be accessed via: http://mialab.ruc.edu.cn/ACPredBMFServer/.

## Supplementary Information

Supplementary Information.

## Data Availability

The authors confirm that the data generated or analysed during this study are included in this article and its supplementary information files. The specific prediction results in this study are available at http://mialab.ruc.edu.cn/ACPredBMFServer/.
