# Supplementary material for: ACPred-BMF: bidirectional LSTM with multiple feature representations for explainable anticancer peptide prediction
Source: Sci Rep. 2022 Dec 19;12:21915. doi: 10.1038/s41598-022-24404-1 (PMC9763336; doi:10.1038/s41598-022-24404-1)
Supplement: Supplementary file 1 — Supplementary Information. [file 41598_2022_24404_MOESM1_ESM.pdf]

ACPred-BMF: Bidirectional LSTM with multiple feature representations for explainable anticancer peptide prediction

Bingqing Han[1], Nan Zhao[1], Chengshi Zeng[1], Zengchao Mu[2,*]& Xinqi Gong[1,3,*]

[1]Institute for Mathematical Sciences, Renmin University of China, Beijing 100872, China

[2]School of Mathematics and Statistics, Shandong University, Weihai 264209, China

[3]Beijing Academy of Artificial Intelligence, Beijing 100083, China

[*] xinqigong@ruc.edu.cn; muzengchao@sdu.edu.cn

Supplementary data S1. Five-fold cross-validation results during model optimization on the main dataset. ‘neurons’ is the number of neurons in the first fully connected layer, and ‘units’ is the number of units in the Bi-LSTM layer.

	epochs	units	neurons	Acc(%)	Sen(%)	Spc(%)	MCC	AUC
1	5	32	50	70.68	73.59	67.76	0.418	0.807
2	15	32	50	73.80	75.76	71.84	0.480	0.832
3	25	32	50	75.04	71.55	78.53	0.505	0.832
4	35	32	50	74.89	73.00	76.78	0.499	0.824
5	45	32	50	73.95	72.72	75.18	0.480	0.823
6	55	32	50	74.24	72.14	76.35	0.486	0.819
7	65	32	50	73.87	72.85	74.89	0.478	0.816
8	5	32	100	69.81	60.82	78.81	0.406	0.802
9	15	32	100	74.74	70.39	79.10	0.498	0.830
10	25	32	100	74.16	71.55	76.78	0.484	0.829
11	35	32	100	74.02	74.31	73.73	0.482	0.829
12	45	32	100	74.97	74.76	75.18	0.501	0.829
13	55	32	100	74.61	72.72	76.49	0.493	0.823
14	65	32	100	74.75	75.05	74.46	0.497	0.823
15	5	64	50	71.62	74.60	68.65	0.437	0.817
16	15	64	50	75.25	76.20	74.31	0.508	0.838
17	25	64	50	75.54	74.60	76.48	0.511	0.837
18	35	64	50	74.60	75.91	73.28	0.494	0.826
**19**	**45**	**64**	**50**	**75.76**	**76.93**	**74.60**	**0.516**	**0.827**
20	55	64	50	75.33	73.59	77.06	0.509	0.834
21	65	64	50	74.60	75.91	73.29	0.493	0.830
22	5	64	100	71.55	75.91	67.20	0.434	0.814

23	15	64	100	74.82	69.38	80.25	0.504	0.839
24	25	64	100	73.00	68.65	77.35	0.464	0.826
25	35	64	100	73.73	70.69	76.79	0.477	0.822
26	45	64	100	73.22	73.15	73.30	0.465	0.813
27	55	64	100	74.53	73.01	76.06	0.491	0.820
28	65	64	100	73.80	70.68	76.93	0.478	0.821
29	5	128	50	71.05	69.80	72.29	0.428	0.810
30	15	128	50	74.46	74.02	74.90	0.490	0.836
31	25	128	50	75.54	75.47	75.62	0.512	0.832
32	35	128	50	74.31	74.46	74.16	0.487	0.825
33	45	128	50	74.46	73.29	75.62	0.490	0.823
34	55	128	50	72.79	73.30	72.28	0.457	0.822
35	65	128	50	74.60	74.32	74.90	0.493	0.829
36	5	128	100	71.40	68.05	74.74	0.435	0.813
37	15	128	100	75.25	71.84	78.67	0.509	0.831
38	25	128	100	73.30	74.17	72.43	0.468	0.820
39	35	128	100	74.53	72.56	76.49	0.492	0.817
40	45	128	100	74.39	74.02	74.75	0.489	0.823
41	55	128	100	74.67	73.74	75.62	0.494	0.818
42	65	128	100	73.80	74.30	73.29	0.480	0.819
43	5	256	50	71.12	67.50	74.74	0.430	0.811
44	15	256	50	74.39	76.77	72.00	0.494	0.822
45	25	256	50	73.87	73.73	74.03	0.478	0.821
46	35	256	50	74.38	73.44	75.33	0.488	0.819
47	45	256	50	73.22	72.86	73.59	0.465	0.815
48	55	256	50	73.95	73.87	74.02	0.479	0.815
49	65	256	50	74.09	73.16	75.04	0.482	0.817
50	5	256	100	71.92	74.17	69.66	0.445	0.810
51	15	256	100	74.89	76.77	73.00	0.502	0.829
52	25	256	100	73.95	72.13	75.77	0.481	0.822
53	35	256	100	73.87	69.23	78.52	0.481	0.821
54	45	256	100	74.74	71.99	77.50	0.496	0.825
55	55	256	100	74.02	72.85	75.18	0.481	0.821
56	65	256	100	74.46	73.88	75.04	0.490	0.821

Supplementary data S2. Five-fold cross-validation results during model optimization on the alternate dataset. ‘neurons’ is the number of neurons in the first fully connected layer, and ‘units’ is the number of units in the Bi-LSTM layer.

	epochs	units	neurons	Acc(%)	Sen(%)	Spc(%)	MCC	AUC
1	5	32	50	89.76	87.11	92.39	0.797	0.962
2	10	32	50	90.21	89.56	90.85	0.805	0.965
3	15	32	50	91.24	90.98	91.49	0.825	0.967

4	20	32	50	90.53	89.05	92.01	0.812	0.965
5	25	32	50	90.79	91.76	89.82	0.816	0.967
6	30	32	50	90.01	88.16	91.88	0.805	0.963
7	35	32	50	90.46	90.46	90.46	0.810	0.964
8	5	32	100	89.82	90.08	89.57	0.798	0.963
9	10	32	100	88.92	93.68	84.15	0.784	0.967
10	15	32	100	90.08	89.18	90.99	0.805	0.969
11	20	32	100	90.33	87.50	93.17	0.810	0.968
12	25	32	100	90.40	90.08	90.72	0.809	0.968
13	30	32	100	90.46	90.99	89.94	0.810	0.966
14	35	32	100	90.98	90.21	91.75	0.821	0.967
15	5	64	50	89.63	90.47	88.79	0.795	0.962
16	10	64	50	90.59	90.34	90.85	0.813	0.969
17	15	64	50	90.40	90.59	90.21	0.809	0.967
18	20	64	50	90.14	90.98	89.30	0.805	0.966
19	25	64	50	90.66	90.21	91.11	0.814	0.967
20	30	64	50	90.91	89.95	91.88	0.819	0.967
21	35	64	50	91.24	91.50	90.98	0.825	0.967
22	5	64	100	89.88	88.28	91.50	0.800	0.964
23	10	64	100	90.01	90.85	89.17	0.802	0.964
24	15	64	100	90.21	88.54	91.88	0.806	0.967
25	20	64	100	90.40	91.75	89.05	0.808	0.965
26	25	64	100	90.33	90.86	89.82	0.808	0.965
27	30	64	100	90.53	90.60	90.46	0.811	0.965
28	35	64	100	90.91	90.98	90.85	0.819	0.966
29	5	128	50	89.69	90.59	88.79	0.795	0.962
30	10	128	50	90.21	89.56	90.86	0.805	0.965
31	15	128	50	90.01	88.79	91.23	0.802	0.964
32	20	128	50	90.59	89.05	92.14	0.813	0.964
33	25	128	50	90.98	90.98	90.98	0.820	0.967
34	30	128	50	91.37	90.72	92.01	0.828	0.968
**35**	**35**	**128**	**50**	**91.49**	**90.85**	**92.14**	**0.831**	**0.968**
36	5	128	100	91.24	90.73	91.75	0.826	0.967
37	10	128	100	90.01	88.79	91.24	0.801	0.965
38	15	128	100	89.82	90.47	89.17	0.803	0.964
39	20	128	100	90.01	89.95	90.08	0.802	0.962
40	25	128	100	90.27	88.54	92.01	0.808	0.962
41	30	128	100	90.21	90.08	90.34	0.805	0.964
42	35	128	100	90.01	89.95	90.08	0.801	0.964
43	5	256	50	89.63	89.69	89.56	0.793	0.960
44	10	256	50	89.43	88.66	90.21	0.789	0.965
45	15	256	50	88.60	90.59	86.60	0.774	0.961
46	20	256	50	89.56	89.56	89.56	0.792	0.961

47	25	256	50	89.75	89.56	89.95	0.796	0.961
48	30	256	50	89.95	90.47	89.43	0.801	0.963
49	35	256	50	89.88	89.56	90.20	0.798	0.962
50	5	256	100	89.24	85.96	92.52	0.789	0.962
51	10	256	100	90.27	89.69	90.85	0.806	0.965
52	15	256	100	90.14	90.98	89.30	0.804	0.965
53	20	256	100	90.21	90.47	89.95	0.804	0.960
54	25	256	100	90.33	89.95	90.72	0.807	0.963
55	30	256	100	90.53	90.08	90.98	0.811	0.962
56	35	256	100	90.66	89.95	91.36	0.814	0.961
